# Carbon stock assessment of Mangrove habitat using field measurements and using machine learning-based GIS and remote sensing techniques

**DOI:** 10.1016/j.isci.2025.114053

**Published:** 2025-11-14

**Authors:** Khaled Al-Jabri, Yaseen Al-Mulla, Ahmad Tabook, Nabil Al-Lawati, Fatma Al Lawati, Ahmed Al Abri, Salma Rabani, Nasser Al Salmi

**Affiliations:** 1 Department of Soils, Water and Agricultural Engineering, Sultan Qaboos University, Al-Khoud 123, Muscat, Oman; 2 Remote Sensing and GIS Research Center, Sultan Qaboos University, Al-Khoud 123, Muscat, Oman; 3 Petroleum Development Oman, Al-Qurum 100, Muscat, Oman

**Keywords:** Global carbon cycle, Remote sensing, Plant ecology, Biogeoscience

## Abstract

Mangrove ecosystems are critical blue-carbon habitats that contribute to climate change mitigation through carbon sequestration. This study assessed aboveground biomass (AGB) and carbon stocks of *Avicennia marina* mangroves in a hyper-arid environment using integrated field and remote sensing approaches. Destructive sampling and field surveys were combined with high-resolution Pléiades *Neo* satellite imagery to calibrate biomass models. A newly developed allometric model achieved high accuracy (R^2^ = 0.93, MAPE = 42.9%), estimating carbon storage at 0.023 t C per tree for diameters of 4.5–13.0 cm. A machine learning-based remote sensing model also performed reliably (R^2^ = 0.643, MAPE = 17.3%), demonstrating the utility of spectral indices and canopy reflectance for biomass prediction. These results highlight the role of mangroves in national and global carbon accounting and provide practical insights for conservation planning, carbon offset frameworks, and standardized biomass assessment methodologies in arid coastal ecosystems.

## Highlights

• Allometric model predicts mangrove biomass/carbon sequestration in arid climates
• Canopy, diameter, and height drive biomass variation in ecosystem engineering
• Remote sensing and ML improve carbon stock mapping in hyper-arid mangrove systems
• Proved mangroves role in CO_2_ reduction and climate-smart ecosystem engineering

## Graphical abstract

**Figure:**
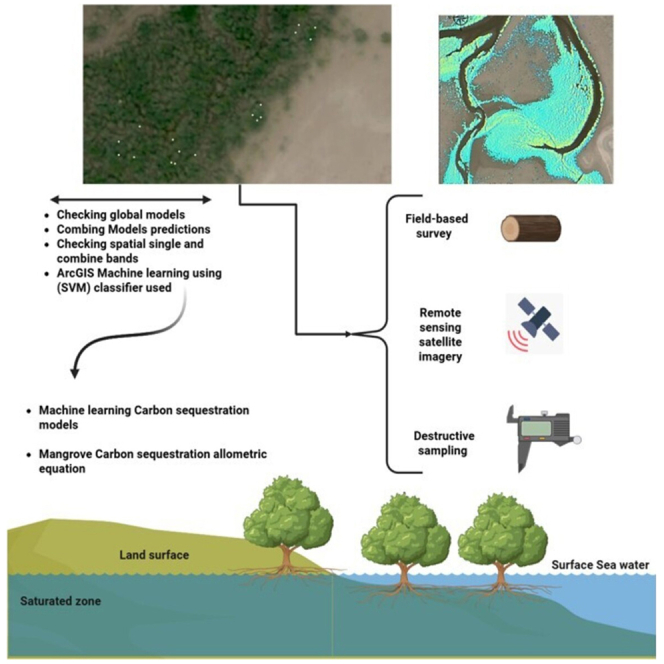

## Introduction

Mangroves are highly productive coastal ecosystems comprising salt-tolerant trees and shrubs. These ecosystems provide a wide range of ecosystem services—classified as provisioning, regulating, cultural, and supporting that directly or indirectly enhance societal well-being.^1^^,^^2^^,^^3^ Although the intrinsic value of ecosystems to human welfare has been recognized for decades, the formal articulation of the term “ecosystem services” gained momentum following the Millennium Ecosystem Assessment in 2005, which significantly shaped the public and policy discourse.^4^

Globally, mangroves span an estimated 12–20 million hectares across 118 countries, with the Food and Agriculture Organization^5^ estimating approximately 15.2 million hectares.^6^ Despite covering just 3% of the world’s forest area, mangroves exhibit a disproportionately high capacity for carbon sequestration often exceeding that of tropical rainforests.^7^^,^^8^^,^^9^^,^^10^

Nevertheless, mangroves have long been undervalued and degraded, particularly prior to the 1980s. Historically, they were often seen as unproductive and unpleasant, which led to their overexploitation and conversion.^11^ This perception began to shift following a 1978 UNESCO initiative that spurred global interest in mangrove conservation. The IUCN subsequently identified several mangrove species,^12^ underlining the urgency for sustainable management practices.

The Omani coastline spans roughly 3,165 km, encompassing coastal islands and borders three distinct water bodies: the Arabian Gulf, Sea of Oman, and Arabian Sea.^13^ Oman is among the 124 nations that harbor mangroves, with an estimated forest area covering 1,100 ha,^14^ and it holds the distinction as the center for mangrove conservation in the Arabian peninsula.^15^ Historical accounts suggest that mangroves once extensively occupied the Omani coastline.^14^ However, today, the area covered by mangroves has considerably reduced.^16^

To evaluate the ecological significance of mangroves, particularly their role in carbon sequestration, there is a pressing need for accurate, spatially explicit estimates of biomass and carbon stock. Conventional field-based methods are labor-intensive and often impractical over large areas. In this context, integrating field measurements with remote sensing and modeling techniques offers a scalable solution.

Understanding descriptive parameters like tree height and trunk diameter is critical for studying the structural attributes of mangroves. Specifically, diameter-at-breast height or stem basal diameter is strongly linked to the development of mangrove stands, allowing estimation of crown spread, basal area, and biomass. These structural characteristics play a crucial role in defining spatial variation and temporal changes in understory vegetation. Accurate estimation of biomass^17^ is essential for describing current mangrove forest conditions and predicting potential impacts of changes. Allometric relationships derived from measuring various structural parameters in a forest enable the estimation of other complex parameters that are challenging to measure directly. Numerous research initiatives have developed allometric equations using diameter-at-breast height measurements collected *in situ* to estimate tree volume and aboveground biomass (AGB).^18^^,^^19^^,^^20^^,^^21^^,^^22^

Precise evaluation of carbon reserves is imperative before initiating any conservation or restoration efforts.^23^ The amalgamation of destructive methods and on-site measurement techniques facilitates a more sophisticated estimation of AGB. The carbon retained within both biomass and sediments in coastal ecosystems is commonly referred to as blue carbon, a term referenced in various studies.^12^^,^^24^^,^^25^ Mangroves, in particular, are recognized as one of the most carbon-dense ecosystems globally. Studies by^26^^,^^27^^,^^28^ underscore the substantial carbon sequestration potential of mangroves and advocate for the development of effective recovery and conservation strategies. The growing interest in the role of mangroves in carbon storage is largely driven by increasing concerns over global warming.^27^^,^^28^ While numerous studies have examined carbon sequestration in mangrove wetlands across regions such as Saudi Arabia,^29^ the United Arab Emirates,^30^ and India,^31^ there remains limited understanding regarding the significance of mangrove wetlands in hyper-arid areas.^32^

Unlike previous studies that relied on generalized allometric equations without direct biomass measurement, this study employed destructive sampling to obtain actual biomass data and develop new site-specific models. Given the influence of localized environmental conditions—such as salinity, sediment type, microclimate, and the unique positioning of Oman’s mangrove stands along the Arabian Sea—this approach offers improved accuracy in estimating carbon stocks and better informs ecosystem-specific conservation strategies. The development of new equations tailored to Al Sawadi’s ecological conditions ensures more precise carbon assessments, particularly in light of the area’s distinct oceanographic and environmental factors that influence mangrove growth and carbon dynamics.

Despite Oman’s endorsement of the United Nations Framework Convention on Climate Change and the 2016 Paris Agreement, the carbon sequestration potential of its natural ecosystems remains largely unexplored. This study aims to (1) quantify the aboveground carbon stock of mangrove stands in Oman using destructive and non-destructive measurements and (2) develop and validate allometric equations integrating field measurements, high-resolution satellite imagery, and machine learning algorithms. The findings are expected to guide carbon-offset policies and contribute to mangrove conservation and climate mitigation strategies.

**Figure 1:**
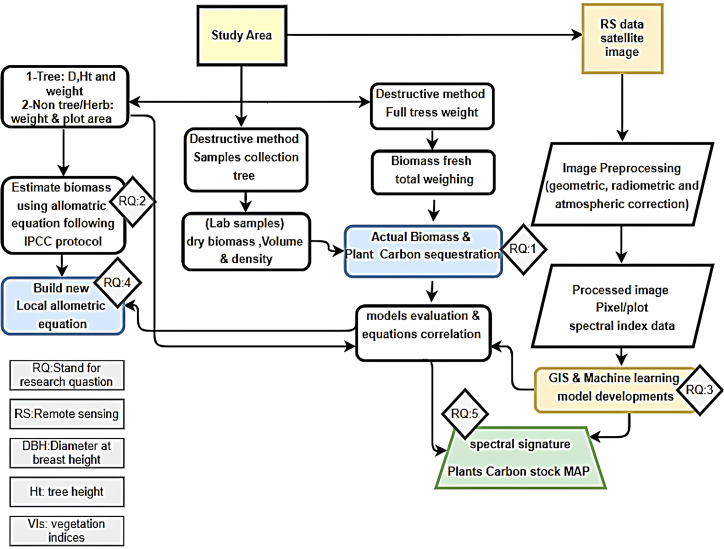
Site field measurements and work stages operational process

Figure 1 presents key parameters commonly considered in assessing biomass and carbon stocks within mangrove ecosystems. In general, structural attributes such as canopy density, species composition, and substrate characteristics influence carbon storage capacity. Mangroves established on finer, organic-rich sediments tend to retain more belowground carbon, while sandy or coarser substrates often limit long-term accumulation through enhanced drainage and reduced nutrient retention. These relationships highlight how biophysical factors collectively shape carbon sequestration potential across mangrove environments and form the conceptual basis for ecosystem-specific modeling frameworks recommended by the IPCC guidelines.^33^^,^^34^

## Results

### In-situ mesaurnets and descrutive sampling

Table 1 exhibits the physical attributes of seven trees, encompassing their respective minimum and maximum height, initial branch depth, stem diameter, and canopy dimensions. These metrics hold pivotal significance in comprehending the physical traits and ecological functionalities of trees within the environment and serve as essential inputs for employing the allometric equation. The range of minimum and maximum height furnishes insights into the tree’s size, a critical determinant shaping its ecological niche and interactions within the ecosystem. Stem diameter stands as a fundamental parameter in evaluating tree biomass and carbon storage.

**Table 1:** List of destructive *Avicennia marina* (mangrove) samples measurements results

Tree No	Ht-min (m)	Ht-max (m)	TD (cm)	TC (m)
1	0.8	1.5	8.435	2.1
2	1.1	1.6	4.488	1.2375
3	0.6	1.4	5.252	1.4725
4	0.85	1.4	5.252	1.3125
5	0.6	1.2	8.276	1.925
6	0.9	1.8	5.296	1.5225
7	2.8	3.7	13.05	3.85

Ht: tree height, TD: diameter, TC: tree canopy.

Table 2 delineates empirical data derived from a study encompassing seven distinct trees, focusing on the fresh weight and dry weight of stems and branches. These measurements hold pivotal significance in comprehending biomass distribution and the growth patterns exhibited by trees. The “full tree fresh weight (kg)” denotes the recorded weight encompassing the entire tree, including stems and branches, offering an insight into the overall size and mass of each tree. Moreover, the study involved the extraction of stem and branch samples from each tree, measuring their respective fresh weights. Additionally, the investigation included determining the dry weights of these stem and branch samples by eliminating moisture content and measuring the remaining weight.

**Table 2:** List of destructive *Avicennia marina* (mangrove) samples measurements results

Tree No	Full tree fresh wt (kg)	Stem sample fresh wt(g)	Branch sample fresh wt(g)	Stem sample dry wt(g)	Branch sample dry wt(g)
1	24.2	410	480	240	260
2	6.62	330	320	190	130
3	6.4	390	340	230	160
4	8.2	510	320	270	160
5	12.2	390	370	220	170
6	10.2	450	400	250	190
7	98.3	530	530	290	260

Wt: weight.

Table 3 presents calculated data encompassing various metrics associated with trees, notably including stem wet-dry ratios, branch wet-dry ratios, average tree wet-dry ratios, tree AGB, and tree AGB carbon stock. The “stem wet-dry ratio” signifies the ratio between the wet weight and dry weight of the stems in each tree, while the “branches wet-dry ratio” represents the analogous ratio for branches, offering insights into their moisture content. Additionally, the “average tree wet-dry ratio” computes the mean wet-dry ratio considering both stems and branches, providing an overall depiction of the moisture content across the entire tree.

**Table 3:** List of destructive *Avicennia marina* (mangrove) sample measurements results

Tree No	stem wet-dry ratio	Branches wet-dry ratio	Average tree wet-dry ratio	Tree AGB biomass (kg)	Tree AGB Carbon stock (kg)
1	0.585	0.542	0.564	13.637	6.819
2	0.576	0.406	0.491	3.250	1.625
3	0.590	0.471	0.530	3.393	1.697
4	0.529	0.500	0.515	4.221	2.110
5	0.564	0.459	0.512	6.244	3.122
6	0.556	0.475	0.515	5.256	2.628
7	0.547	0.491	0.519	51.005	25.502

Moreover, the “tree AGB biomass (kg)” showcased in Table 3 denotes the AGB of individual trees, measured in kilograms using a destructive method. This metric represents the collective mass of living vegetation above the ground, encompassing stems, branches, leaves, and other aboveground components. Lastly, the “Tree AGB Carbon stock (kg)” quantifies the quantity of carbon stored within the AGB of each tree, expressed in kilograms. Tree 7, which appears to exhibit large deviations in several parameters, represents the largest individual sampled and contributed disproportionately to total biomass. This variation reflects the natural range of tree sizes within the ecosystem and provides valuable input for capturing the upper bounds of biomass in the model.

**Figure 2:**
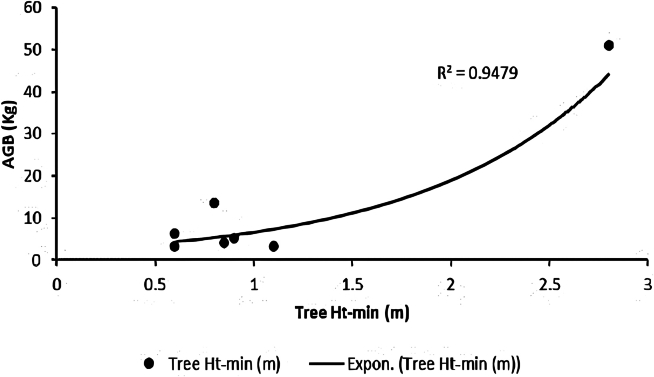
Destructive sampling of *Avicennia marina* (mangrove): calculated actual above ground biomass (kg/tree) versus tree at minimum height (m)

**Figure 3:**
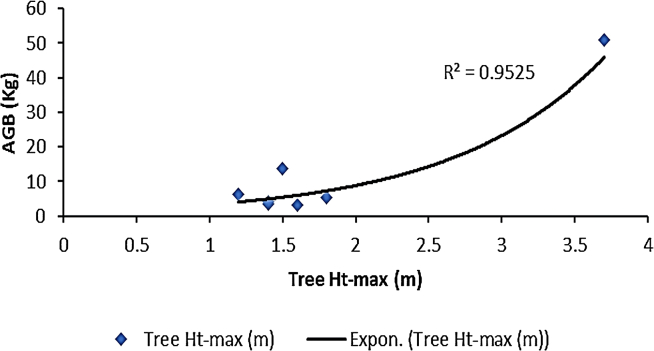
Destructive sampling of *Avicennia marina* (mangrove): calculated actual above ground biomass (kg/tree) versus tree maximum height (m)

**Figure 4:**
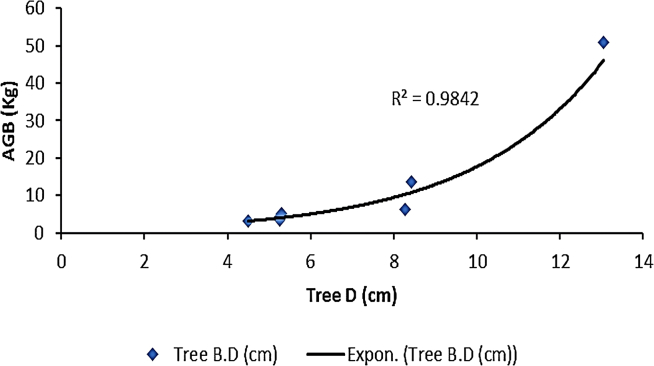
Destructive sampling of *Avicennia marina* (mangrove): calculated above ground biomass (kg/tree) versus tree diameter (cm)

**Figure 5:**
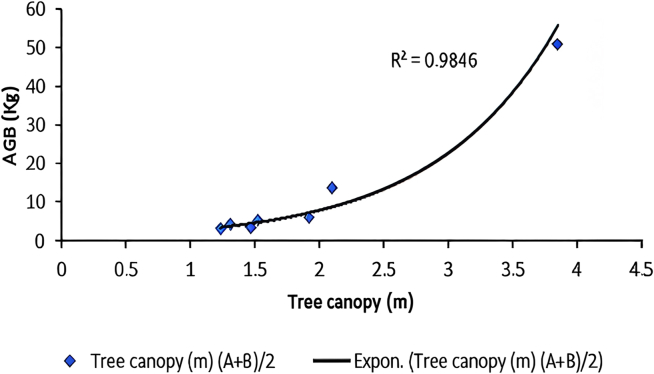
Destructive sampling of *Avicennia marina* (mangrove): calculated above ground biomass (kg/tree) versus tree canopy (m)

Figures 2, 3, 4, and 5 delineate the R2 exponential trendline values derived from the assessment of calculated AGB concerning the recorded dimensions of mangrove trees, encompassing attributes such as the tree’s min-max height, diameter, and computed tree density. These visual representations depict the observed R2 values, revealing a promising trend that signifies a significant correlation between the estimated AGB and the measured tree attributes. To support and validate these findings, *in situ* measurements of 160 trees were conducted in addition to destructive sampling. These included detailed records of tree height and diameter at breast height (DBH), which were critical for refining the allometric equations used in biomass estimation. An exponential trendline was selected over linear alternatives as it consistently provided a higher coefficient of determination (R^2^), aligning with ecological patterns where biomass tends to accumulate at an accelerating rate with tree growth. However, it remains crucial to augment and validate these findings by incorporating additional measurements of tree parameters acquired directly from the study site. This comprehensive approach is pivotal for conducting meticulous comparisons and establishing correlations of the obtained outcomes within the broader context of the entire study area.

The findings revealed an AGB carbon stock of 2.43 kg/tree (3.61 kg/m^2^) and carbon sequestration of 6.2 kg C/tree (1.81 kg C/m^2^). The optimal model demonstrated R^2^ = 0.98 and root mean-square error (RMSE) = 4.55.

Table 4 compares our study findings of the carbon stock measurements of *Avicennia marina* with previously reported findings in various locations of the world that shared similar climate to Oman. In this study, carbon stock in Oman’s Al Sawadi area was assessed, with an average of 12.4 kg per tree (3.61 kg/m^2^) and an estimated 1.81 kg/m^2^ of carbon stock. Abohassan^35^ measured 1.47 kg/m^2^ in KSA, Mashaly^36^ found 13.0 kg/m^2^ in Egypt,^19^ reported 1.7 kg/m^2^ in Iran, and Dodd^37^ noted 9.0 kg/m^2^ in the UAE. Alsumaiti^38^ recorded 1.3 kg/m^2^ in the UAE, while^22^ observed an average of AGB of 6.5 kg/m^2^ in seaward in Oman’s Alqurum. Al-Guwaiz^29^ measured 1.39 kg/m^2^ in Yanbu, 0.88 kg/m^2^ in Ar-Rayis, and 0.806 kg/m^2^ in Umluj, all in KSA. Rovai^39^ documented 1.73 kg/m^2^ in fringe forest and 3.02 kg/m^2^ in interior forest in Brazil, while Kairo^40^ reported 1.17 kg/m^2^ in Kenya. These varied measurements highlight regional differences in mangrove carbon storage.

**Table 4:** Productivity of *Avicennia marina* (mangrove) in natural stands in different regions of the world

AGB	Location	Reference	Method
3.61 kg/m^2^	Oman	This Study	Integration of 8 destructive trees/*in situ* measurement
1.47 kg/m2	KSA	Abohassan et al.^35^	Allometric equation
13.0 kg/m2	Egypt	Mashaly et al.^36^	Allometric equation
1.7 kg/m2	Iran	Parvaresh et al.^19^	8 destructive trees
9.0 kg/m2	UAE	Dodd et al.^37^	Hydrocarbons extraction
1.3 kg/m2	UAE	Alsumaiti^38^	Allometric equation
6.5 kg/m2	Oman	Al-Nadabi et al.^22^	Allometric equation
0.81–1.39 kg/m2	KSA	Al-Guwaiz et al.^29^	Three quadrats of 0.5 × 0.5 m were destructive
1.17–3.02 kg/m2	Brazil	Rovai et al.^39^	Allometric equation
1.17 kg/m2	Kenya	Rovai et al.^39^	10 destructive trees

### Statistical analysis

Table 5 summarized the correlation of linear relationship with its value (a, b, and R^2^). The table provides insights into which model exhibits a closer agreement with the actual harvest’s AGB. Among the models, AGBPaul yielded the closest results, while AGBNadabi showed higher values, and AGBConti1 produced lower AGB values while using the diameter predictor. Despite AGBFu showing a strong relationship with plant predictors such as height, diameter, and canopy, its correlation with observed harvested AGB values was weaker due to variations in the a (slope) and b (intercept) values mentioned in Table 5. These indications were considered during the development of the final model.

**Table 5:** The list of *Avicennia marina* (mangrove) AGB logarithms correlated with the logarithms measured tree predictors (diameter, height, and canopy) among actual destructive methods of different studies

Equation	Factor	Slope (a)	Intercept (b)	R^2^	Correlation
LnAGB1	Diameter	2.4873	−2.7719	0.8952	High
	Height	2.1542	+0.8662	0.6536	Medium
	Canopy	2.4577	+0.5651	0.946	High
LnAGB2	Diameter	2.473	−2.6793	1	Extremely High
	Height	1.5831	+1.2272	0.3989	Very Low
	Canopy	2.3251	+0.7067	0.9568	High
LnAGB3	Diameter	2.2991	−1.7289	1	Extremely High
	Height	1.472	+1.903	0.399	Very Low
	Canopy	2.1616	+1.4191	0.9567	High
LnAGB4	Diameter	2.6965	−3.7703	0.9576	High
	Height	2.1664	+0.2613	0.6016	Medium
	Canopy	2.6427	+0.1402	0.9955	High
LnAGB5	Diameter	2.7024	−3.3497	0.9942	High
	Height	1.8733	+0.845	0.465	Low
	Canopy	2.5817	+0.3268	0.9822	High
LnAGB6	Diameter	2.6581	−2.8078	0.8383	High
	Height	2.6054	+0.9229	0.7839	High
	Canopy	2.722	+0.7031	0.9515	High

LnAGB1: Observed in this study from destructive sampling; LnAGB2^41^; LnAGB3^22^; LnAGB4^42^; LnAGB5^42^; LnAGB6.^43^

**Figure 6:**
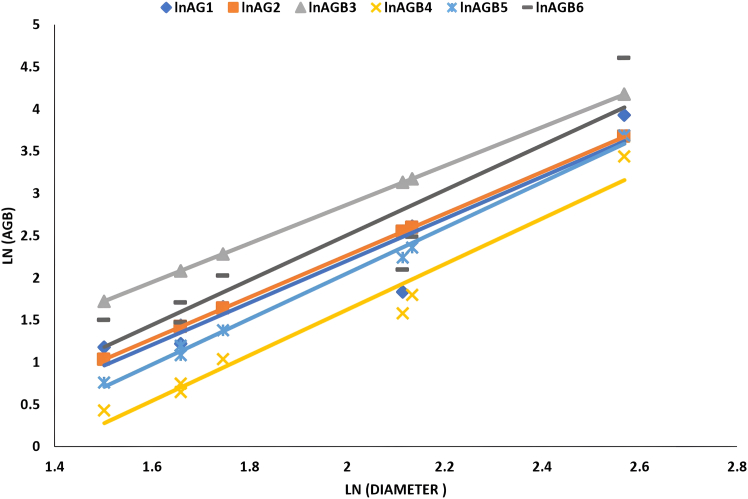
Correlation of *Avicennia marina* (mangrove) AGB vs. Log plant diameter measured of destructive sampling along different evaluated equations

**Figure 7:**
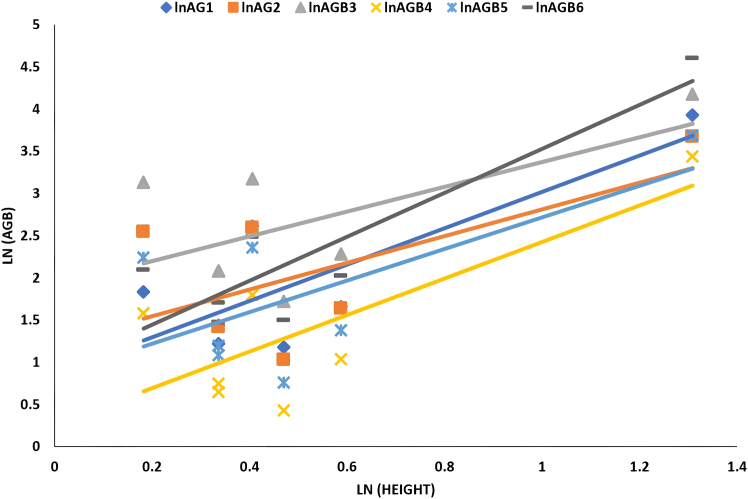
Correlation of *Avicennia marina* (mangrove) AGB log’s vs. log plant height of destructive sampling along different evaluated equations

**Figure 8:**
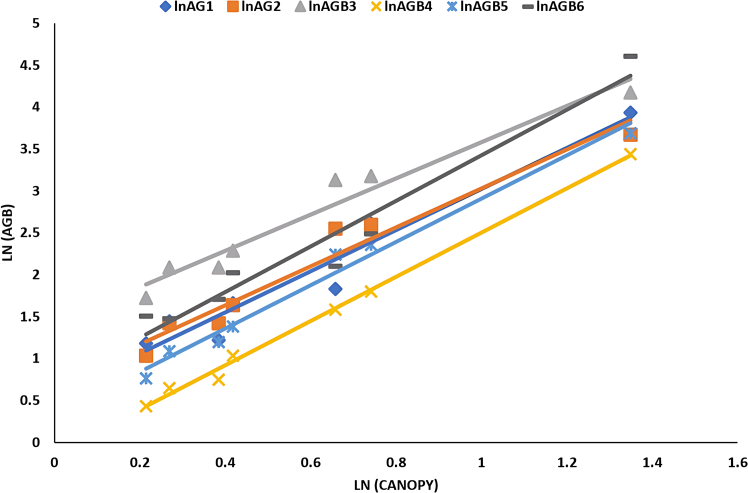
Correlation of *Avicennia marina* (mangrove) AGB log’s vs. log plant canopy of destructive sampling along different evaluated equations

The linear relationship exhibited high R^2^ values with most of the evaluated equations (listed in Table 5), as depicted in Figures 6, 7, and 8. However, the AGB relationship with plant height was only significant when utilizing the equation developed by Fu,^43^ where an R^2^ value of 0.7839 was obtained as shown in Figure 7.

### Developed allometric models

**Figure 9:**
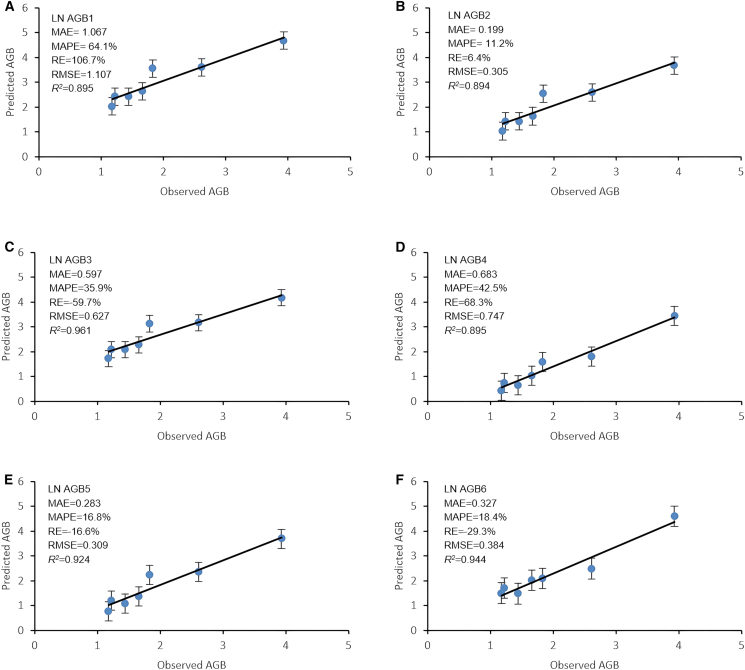
Goodness of fit of actual AGB compared with the four selected models for predicting the aboveground biomass of mangrove trees (*n* = 7) Vertical bars for standard error.

Figure 9 compares the performance of the six tested existing global and local models in predicting AGB of destructive tree samples. AGB2 performed best, with the lowest errors (MAPE: 11.2%, MAE: 0.199, and RMSE: 0.305) and a good correlation (*R*^*2*^: 0.894). AGB5 ranked second, showing slightly higher errors (MAPE: 16.8%, MAE: 0.283, and RMSE: 0.309) but a better correlation (*R*^*2*^: 0.924). AGB5 showed similar output correlations of AGB5. AGB4 had the strongest correlation (*R*^*2*^: 0.961) but higher errors (MAPE: 35.9%, MAE: 0.597, and RMSE: 0.627). AGB1 and AGB3 showed the weakest performance, with high errors and the same correlation.

Initial investigations were conducted using individual predictor variables, specifically tree canopy (CD), diameter (D), and tree height (H) in order to determine which variable provided a more robust explanation for AGB. The selection of correlated fit R^2^ values among the different studies was based on their relevance to the environmental and climatic conditions resembling those found in Oman. In the case of a single linear equation utilizing tree diameter, it is preferable to choose a value of R^2^ that is closer to 1. However, when multiple variables such as height, diameter, and canopy are involved, it is advisable to sequentially consider the studies of Conti equation 2, Conti equation 1^42^ and Fu^43^ based on their closer R^2^ values and *p* values and average AGB (kg/tree) values of destructive sampling comparison (10.34,7.22 and 20.33). Furthermore, Paul allometric equation shows an average AGB (11.69 kg/tree) fits very close to the results of our study average destructive sampling (12.43 kg/tee) and can considered as fit to our study destructive. Notably, another study^22^ which took place in a climate similar to our own study, exhibit a particularly close fit to the tree diameter but with slightly higher AGB values than destructive sampling.

Each dot in Figure 9 represents an individual LnAGB (kg): (a) predicted (AGB1) and observed AGB(ActualAGB) values; (b) predicted (AGB2) and observed AGB (ActualAGB) values; (c) predicted (AGB3) and observed AGB (ActualAGB) values; (d) predicted (AGB4) and observed AGB (ActualAGB) values; (e) predicted (AGB5) and observed AGB (ActualAGB) values; and (f) predicted (AGB6) and observed AGB (ActualAGB) values.

The R^2^ and *p*-value indicated the strength of correlation between LN (AGB) and three or fewer variables. To enhance the regression equations, stepwise modeling was employed, systematically removing variables that lacked significant relationships, thereby ensuring improved accuracy and quality of the obtained result. Stepwise modeling was utilized to improve the regression equations by eliminating variables that were not significantly related, ensuring better quality of the results. The statistical software Minitab 21.1.0 was utilized to perform data modeling analysis with stepwise regression analysis features. This method, known as an iterative approach, adds or removes predictors based on their statistical significance, typically using an alpha criterion for *p*-values to determine inclusion or exclusion. This method systematically constructs a regression model by iteratively selecting independent variables, adding or removing them, and evaluating their statistical significance after each iteration.

Four novel models were constructed for the mangrove stand by incorporating equations from previous studies,^22^^,^^42^^,^^43^ regression model equations for destructive plant correlation and applying stepwise regression on the data collected from 160 measured plots. The performance of these new models was evaluated using indicators such as R^2^, RMSE, fit statistics, and *p*-Value. The indicators of three variables (D, CD, or H) were included as part of the model. These developed four novel models (Model#lAGB, Model#2AGB, Model#3AGB, Model#4AGB, and the Model#5AGB destructive-driven model) as followings:

(Equation 1) $$\;\text{Model}\#1AGB=e^{\left(-1.7272+0.0007\;\ln\;CD+0.00105\;\ln\;H+2.2977\;\ln\;D\right)}$$

(Equation 2) $$\;\text{Model}\#2AGB=e^{\left(-2.2459+0.8605\;\ln\;CD+0.51613\;\ln\;H+1.4995\;\ln\;D\right)}$$

(Equation 3) $$\;\text{Model}\#3AGB=e^{\left(-2.0605+0.9411\;\ln\;CD+0.00223\;\ln\;H+1.7434\;\ln\;D\right)}$$

(Equation 4) $$\;\text{Model}\#4AGB=e^{\left(0.6045+2.0031\;\ln\;CD+0.99900\;\ln\;H-0.00227\;\ln\;D\right)}$$

(Equation 5) $$\;\text{Model}\#5AGB\;\left(destructive\_driven\right)=e^{\left(0.565+2.458\;\ln\;CD\right)}$$

**Figure 10:**
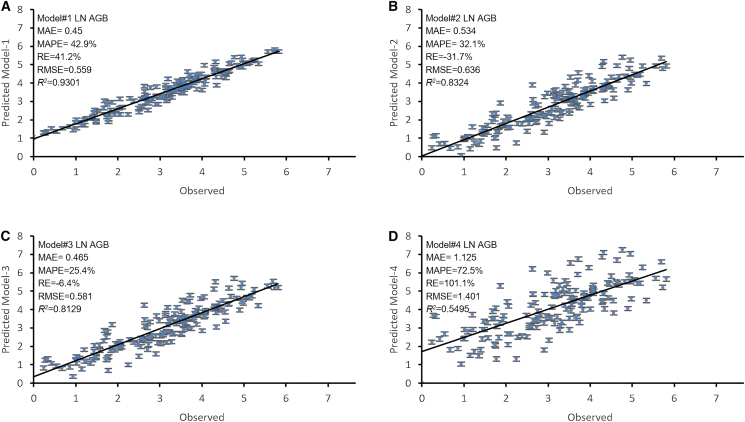
Goodness of fit of model-observed actual AGB using the four driven regression models for predicting the aboveground biomass of *Avicennia marina* (mangrove) (*n* = 160) Vertical bars for standard error.

**Figure 11:**
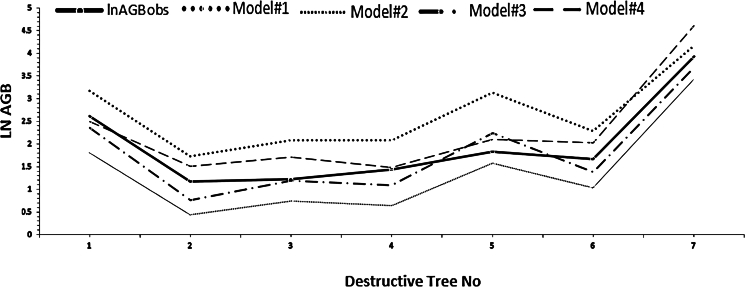
Correlation of *Avicennia marina* (mangrove)-developed novel models of AGB (*n* = 160) versus the corresponding model-observed actual tree AGB (*n* = 7)

**Figure 12:**
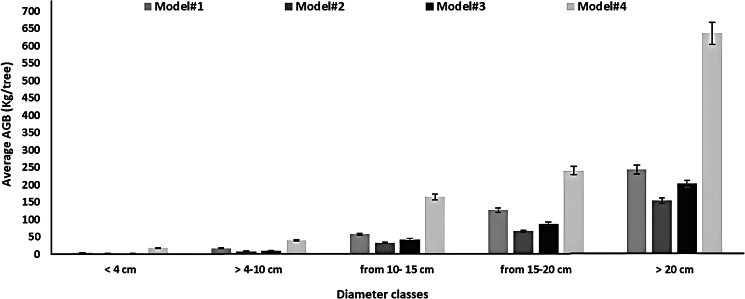
Frequency distribution of mangrove aboveground biomass (AGB) concerning diameters across various models

Upon re-evaluating the newly developed models using destructive values and regression performance metrics (MAPE, MAE, R2, RMSE, and RE) in full measurement trees (*n* = 160), Model-1 demonstrated the strongest agreement (highest *R*^*2*^ and lowest errors) with the observed AGB, followed by Model-3 and Model-2 where they showing the lowest relative bias (RE) fitness. By contrast, Model-4 exhibited the poorest fit to the ActualAGB, showing the lowest performance among all of the developed models (Figures 10; 11; 12).

The black solid line corresponds to a 1:1 relationship. Each dot represents an individual LnAGB (kg): (a) predicted (Model-1) and observed AGB (model-observed actual) values; (b) predicted (Model-2) and observed AGB (model-observed actual) values; (c) predicted (Model-3) and observed AGB (model-observed actual) values; and (d) predicted (Model-4) and observed AGB (model-observed actual) values.

The distribution of diameter class exhibited variations across various models, signifying an uneven age stand structure. This observation aligns with findings regarding *Avicennia marina* forests in Oman’s Al-Qurum Nature reserve,^22^ suggesting ongoing active regeneration within these populations.

Nonetheless, the age of the Al Sawadi reserve, which stands at 20 years, differs from the older trees observed in the Al-Qurum Nature reserve. The results show an indication of better results on the models results with bigger size of tree diameters.

The findings of this study reveal that a minor proportion, specifically 4.46% of the measured stems, possess a diameter below 4 cm. Nearly half of the stems (46.50%) fall within the 4–10 cm diameter range, followed by 29.30% within the 10–15 cm range, 13.38% within the 15 to 20 cm range, and merely 6.4% of the measured stems display a diameter exceeding 20 cm. Research conducted by Al-Nadabi^22^ discovered a substantial presence of trees with diameters exceeding 20 cm within the Al-Qurum Nature reserve in Oman.

**Figure 13:**
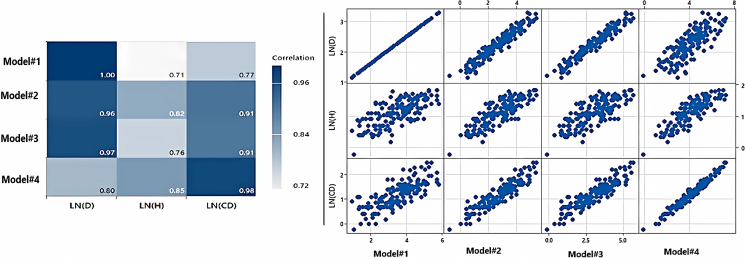
Matrix plots and R^2^ Correlations of the novel models versus the tree measured predictors

The final selection of each model depended on several factors, including the (1) Oasis study, (2) tree’s age, (3) climatic conditions, (4) availability of tree indicators, and (5) destructive measurements (as shown in Table 6). Considering these factors, we were able to determine the most suitable model from the four models. The models were visually represented in a matrix view, depicting the correlation between the measured trees (*n* = 160) and each tree predictor (diameter, canopy, or height) (Figure 13). When combining multiple predictors, the models could either strengthen or weaken based on their R^2^, RMSE, and fit statistics. In this scenario, the stepwise regressions were the preferred option, as they offer a systematic approach to select the most relevant predictors, enhancing the model’s overall performance. After reevaluating the new models using the destructive values, the models that exhibit a closer alignment with the calculated actual destructive biomass, in the given order, were Models 3, 2, 1, and 4 (Table 6).

**Table 6:** Performance of *Avicennia marina* (mangrove) AGB models Fitness

Model	Models correlation fitness to destructive sampling (R^2^)	Fitness to destructive sampling (*n* = 7)			Fitness to all field measurements (*n* = 160)		Fitness level
		Average AGB Kg/tree^a^	Average AG carbon stock Kg/tree	Average AG carbon dioxide sequestered t C/Tree	Average AGB Kg/tree^b^	Average AG carbon dioxide sequestered t C/tree	Recommended models’ level^c^
Model-1	0.90	20.47	10.23	0.037	57.23	0.11	Fits low
Model-2	0.98	7.23	3.61	0.013	32.2	0.059	Fits good
Model-3	0.96	10.33	5.17	0.019	41.83	0.078	Fits high
Model-4	0.97	20.33	10.16	0.037	140.42	0.258	Very low

a Average values from destructive method = 12.43 kg/tree.

b Average values from field measurements = 45.16 kg/tree.

c Based on fitness to all field measurements (*n* = 160).

On the other hand, these differences indicated a consistent pattern where the results became increasingly similar as the trees transition from shrub level to tree level. When evaluating individual predictors (diameter, canopy, or height) based on R^2^ values, Model-1 was the most effective for the diameter predictor, while the model-4 demonstrated better performance for the height and canopy predictors if only one variable is used in the model.

### Machine learning-based remote sensing and GIS techniques

**Figure 14:**
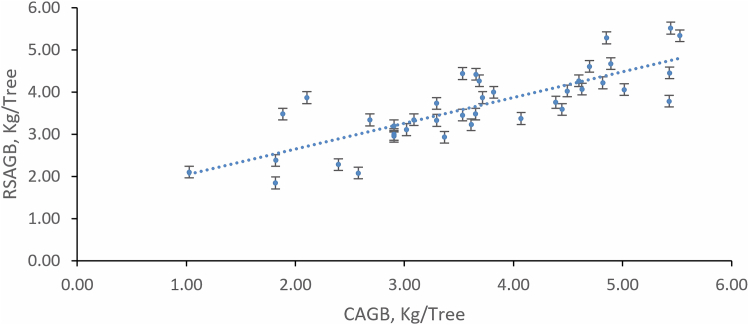
A 1:1 graph of linear regression between predicted remote sensing above ground biomass (RSAGB) vs. calculated above ground biomass (CAGB) of Model#4

The results of the remote sensing above ground biomass (RSAGB) with the tree canopy AGB (CAGB) were correlated using the four different models for the mangrove trees. The analysis showed that Model-4 provided significant results with the highest R2 value of 0.643. Figure 14 shows the correlation between the CAGB and the RSAGB. The analysis proved that the RSAGB can provide accurate results when compared with the CAGB using Model-4 (Table 7).

**Table 7:** Mangrove goodness of fit metric indicators of built-observed actual CAGB models (Model 3 & Model 4) versus RSAGB corresponds to a 1:1 relationship

Metric indicators	(M#3)-Value	(M#4)-Value
R^2^ (R-squared correlation)	0.519	0.643
MAE (Mean Absolute Error)	0.678	0.510
RMSE (Root-Mean-Square Error)	0.829	0.676
Relative bias in percent	−0.7%	0.3%
MAPE (Mean Absolute Percentage Error)	42.4%	17.3%

**Figure 15:**
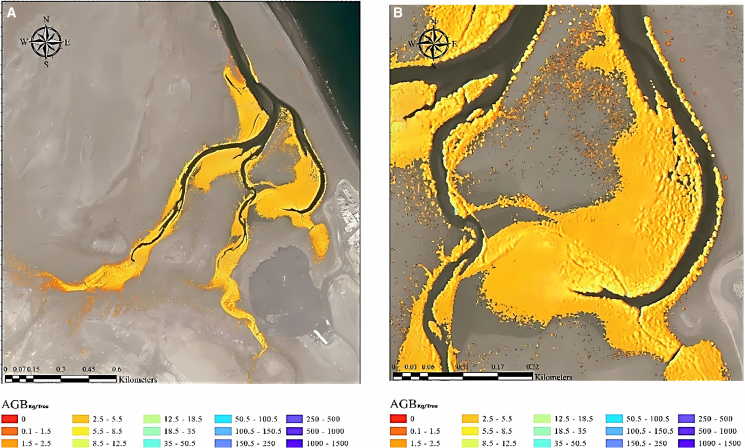
RS-based AGB (Kg/tree) estimated using satellite imagery for the mangrove plants (A) overall imagery, (B) zoomed imagery.

Detailed maps depicting the RSAGB for mangrove vegetation were methodically generated utilizing satellite imagery. These maps were instrumental in visualizing and analyzing the distribution patterns of RSAGB across the mangrove tree species. As illustrated in Figure 15, an overlay technique was employed to exhibit the RSAGB data superimposed upon the identified locations of detected mangrove trees, offering a pixel-by-pixel representation. This visual overlay provided a comprehensive depiction of the spatial relationship between RSAGB distribution and the specific spatial arrangement of the identified mangrove trees within the satellite imagery. This mapping approach facilitated a nuanced exploration of the correlation between RSAGB patterns and the distribution dynamics of mangrove trees, thereby contributing to a deeper understanding of the biomass distribution across the mangrove population.

**Figure 16:**
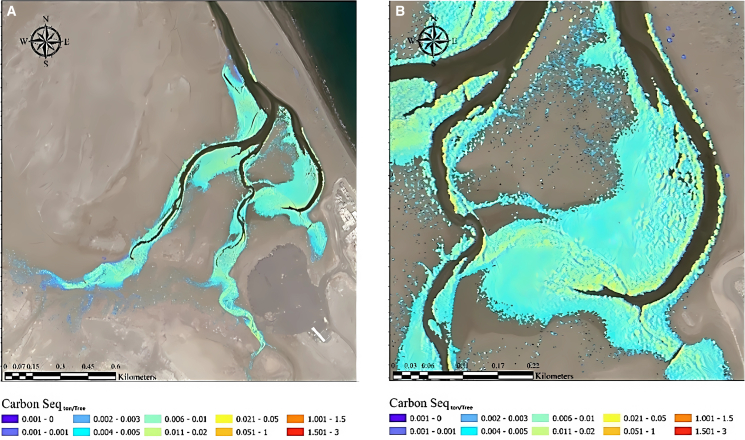
RS-based carbon-Seq. (ton/Tree) estimated using satellite imagery for the mangrove plants (A) overall imagery, (B) zoomed imagery.

A comprehensive cartographic representation delineating carbon sequestration region, derived from satellite data in conjunction with a machine learning algorithm (RS-Carbon-Seq.), was also generated. These detailed maps were specifically designed to visualize and analyses the spatial distribution of areas involved in carbon sequestration. Illustrated in Figure 16 is the superimposition of RS-Carbon-Seq. data onto the detected locations of mangrove trees at a pixel-level resolution. RS-Carbon-Seq. refers to the integration of remote sensing and carbon sequestration estimation techniques, where a support vector machine (SVM) classifier was used to isolate mangrove canopy reflectance and remove background noise. Zonal statistics were applied to extract the mean spectral values per sampling plot, which were then correlated with ground-measured biomass data. These variables, combined within a GIS framework, supported machine learning-driven models to estimate biomass and carbon stock at high spatial precision. This visual representation facilitates an in-depth examination of the relationship between the areas identified for carbon sequestration using RS-Carbon-Seq. and the specific spatial arrangement of detected mangrove trees within the satellite imagery. Such mapping endeavors contribute significantly in comprehending the association between carbon sequestration zones and the presence of mangrove trees within the area studied.

## Discussion

This study aimed to assess the AGB and carbon storage of mangrove trees within the Al Sawadi Reserve in Oman. Through destructive sampling of 160 full measurement trees, we generated essential data that enabled precise calculation of carbon storage across the study site. By combining this field data with various allometric equations and statistical models, we quantified carbon stocks at both tree and ecosystem scales, thereby gaining valuable insights into the carbon sequestration capacity of these ecologically significant habitats.

One of the key methods applied was the use of allometric equations that relate tree structural parameters—such as height, diameter, and canopy diameter—to biomass. These equations are well-established in forestry science and have demonstrated high utility in biomass estimation. Our findings supported the validity of this approach, yielding AGB estimates that were consistent with those from similar mangrove studies conducted in Egypt, Brazil, and Saudi Arabia.^35^^,^^36^^,^^40^ Nevertheless, notable differences in regional carbon densities were observed, highlighting the influence of local environmental conditions.

Specifically, the average carbon stock in the Al Sawadi mangrove area was estimated at 1.81 kg/m^2^. This figure aligns with prior regional assessments, such as the one by^22^ in Al-Qurum Reserve. However, it was considerably lower than values recorded in Egypt, where carbon stocks reached up to 13.0 kg/m^2^.^36^ These disparities underscore the role of site-specific environmental variables—such as soil salinity, sediment texture, tidal dynamics, and climate—in shaping carbon storage potential.

Beyond environmental differences, anthropogenic pressures and management history also influence carbon dynamics. In Al Sawadi, urban encroachment, reduced water quality, and insufficient restoration practices have likely limited biomass accumulation.^14^^,^^44^^,^^45^^,^^46^^,^^47^ This emphasizes the need to consider both natural and human-induced factors when interpreting carbon-stock variability across mangrove ecosystems.

The comparative evaluation of developed allometric models further illuminated the impact of model structure and predictor selection on biomass estimation accuracy. Among the four newly developed models, Model 1 (AGBPaul), which incorporated tree diameter, canopy, and height, achieved the strongest alignment with destructively observed AGB, as reflected in its high R^2^ and lowest prediction errors (MAPE, MAE, RMSE, and RE). Model 3 and Model 2 followed in performance, with slightly higher relative error but still acceptable levels of predictive reliability. In contrast, Model 4 performed the poorest, indicating a suboptimal fit for the dataset. These rankings were substantiated by quantitative model evaluation using the full tree measurement dataset (*n* = 160), thus strengthening the robustness of our findings.

The observed variations in intercept and slope values across the models—particularly in different ecological and climatic contexts—significantly influenced model performance. These differences highlight the importance of region-specific calibration of allometric models to ensure their reliability in diverse mangrove environments. Tailoring model parameters to local site conditions can reduce predictive uncertainty and enhance the effectiveness of carbon estimation methods.

The integration of remote sensing techniques provided further depth to this study. RSAGB showed strong spatial correlation with tree canopy-derived AGB, especially when Model 3 was applied. Machine learning-based mapping (RS-Carbon-Seq) successfully delineated spatial variation in carbon sequestration zones, offering actionable insights for ecosystem management. These technologies enable the monitoring of large and inaccessible mangrove stands, thus facilitating targeted conservation and restoration efforts.

Another critical factor observed was the distribution of tree diameters. Most stems measured between 4 and 10 cm, indicating a relatively young stand structure. Only a small fraction exceeded 20 cm, suggesting that Al Sawadi’s mangroves, estimated at 20 years of age, are still in a maturation phase. This age profile is consistent with findings from the Al-Qurum Reserve and explains the relatively lower AGB observed in this site compared to older mangrove forests elsewhere.

Statistical analysis reinforced the advantage of using locally derived models over generic ones. Models incorporating localized environmental parameters achieved higher accuracy, particularly Model 1, which should be considered the best fit for the Al Sawadi Reserve. The study confirms the value of destructive sampling for model calibration and highlights the synergistic potential of integrating field data, remote sensing, and machine learning to support mangrove carbon assessments and ecosystem management.

This study confirms the feasibility of integrating field-based destructive sampling with remote sensing and machine learning to estimate AGB and carbon stocks in arid mangrove ecosystems. The high correlation observed between ground-measured biomass and remote sensing–derived spectral indices—especially NDVI and canopy diameter (R^2^ = 0.946)—validates the reliability of remote estimation in sparse, dryland environments. Model 3, combining SVM classification and zonal statistics, yielded an R^2^ of 0.550 when compared to CAGB values, demonstrating moderate predictive power while maintaining consistency with destructive sampling results (*n* = 7) and field measurements (*n* = 160).

Furthermore, the resulting RS-Carbon-Seq. maps enabled spatial visualization of carbon stock distribution at the pixel level, highlighting areas of high and low sequestration potential. These maps offer actionable data for targeting conservation efforts, prioritizing restoration zones, and informing carbon offset strategies.

The successful application of this integrated approach in hyper-arid conditions underscores its potential scalability to other coastal environments with similar data limitations. This provides a valuable foundation for climate change mitigation by enabling data-driven decision-making and long-term monitoring of blue carbon ecosystems.

This study employed destructive sampling and remote sensing techniques to evaluate the carbon storage and sequestration potential of *Avicennia marina* in the Al Sawadi Reserve. A strong correlation was observed between aboveground carbon and tree diameter, confirmed by geostatistical analysis and robust regression metrics. Among the newly developed allometric models, Model 1 demonstrated the best performance with an R^2^ of 0.98 and a low RMSE of 4.55, indicating its high reliability in predicting biomass for the study area. Field-based measurements showed significant relationships with spectral vegetation indices, where canopy diameter exhibited the strongest correlation (R^2^ = 0.946), followed by diameter (R^2^ = 0.8952) and height (R^2^ = 0.6536). The estimated carbon stock of 1.81 kg/m^2^ was consistent with previous findings in Oman’s Al-Qurum Reserve but lower than those in Egyptian mangroves, which was attributed to the relatively young age of the trees and regional ecological conditions. The integration of field measurements with satellite-derived biomass (RSAGB) and machine learning (RS-Carbon-Seq) allowed for the generation of detailed carbon sequestration maps, improving spatial understanding of carbon distribution across the mangrove ecosystem. Notably, statistical analysis showed that Model 4 produced the highest R^2^ value of 0.644 for RSAGB versus CAGB, and its average estimated carbon dioxide sequestered closely matched both the destructive sampling data (*n* = 7) and all field-based measurements (*n* = 160), highlighting the model’s reliability. These results validate the use of remote sensing as a powerful tool for monitoring mangrove biomass and confirm the utility of site-specific allometric models in arid and hyper-arid regions. The findings also reinforce the critical role of mangroves in climate mitigation, urging the implementation of sustainable management strategies to protect and enhance these ecologically valuable coastal ecosystems.

Nevertheless, the generalizability of the results is limited by several factors. This study was confined to a single hyper-arid site, and it did not incorporate critical environmental variables such as soil type, water availability, or microclimatic conditions, which can influence biomass accumulation and carbon dynamics. The exclusion of belowground biomass and soil organic carbon further limits the comprehensiveness of carbon stock estimates. These constraints may affect the transferability of the models to other ecological contexts.

Future research should aim to overcome these limitations by incorporating broader environmental datasets, expanding destructive and non-destructive sampling across diverse mangrove regions and integrating belowground carbon pools. Additionally, establishing permanent monitoring plots and applying machine learning models across different vegetation types and climates will support more accurate, scalable, and policy-relevant carbon accounting. Conducting similar studies in subtropical, tropical, and coastal environments will also enhance understanding of variability and improve the robustness of carbon sequestration models at regional and global levels.

### Limitations of the study

The complexity of mangrove ecosystems, coupled with the challenges of scaling destructive sampling and remote sensing techniques, limited the scope of this study to specific hyper-arid zones, potentially affecting the generalizability of the results to other regions. While robust correlations were achieved using satellite data and machine learning, the resolution of remote sensing imagery may not capture localized variations in biomass. Additionally, the study focused solely on aboveground carbon, excluding belowground storage in soils and roots, which are critical components of total carbon stock. Temporal limitations further restricted the analysis to short-term observations, overlooking long-term changes in carbon sequestration potential. Despite these constraints, the study provides a valuable framework for advancing carbon stock assessments and highlights the importance of mangroves in hyper-arid zones, offering a foundation for future, more comprehensive investigations.

## Resource availability

### Lead contact

Further information and requests for resources and reagents should be directed to and will be fulfilled by the lead contact, Yaseen Al-Mulla (yalmula@squ.edu.om).

### Materials availability

This study did not generate new materials.

### Data and code availability

• All the data reported in this paper will be shared by the lead contact upon request.
• This paper does not report original codes.
• Any additional information required to reanalyze the data reported in this paper is available from the lead contact upon request.

## Acknowledgments

The authors would like to thank Petroleum Development of Oman for their cooperation and financial support as well as the 10.13039/501100004351Sultan Qaboos University, Oman for logistics support and in-kind contributions that were provided under grant code number CR/DVC/GISC/22/03.

## Author contributions

Conceptualization, K.A.-J. and Y.A.-M.; methodology, K.A.-J., Y.A.-M., and A.T.; investigation, K.A.-J., Y.A.-M., and A.T.; writing – original draft, K.A.-J. and Y.A.-M.; writing – review and editing, K.A.-J., Y.A.-M., A.T., N.A.-L., F.A.L., A.A.A., S.R., and N.A.S.

## Declaration of interests

The authors declare no competing interests.

## STAR★Methods

### Key resources table

**Table:** 

REAGENT or RESOURCE	SOURCE	IDENTIFIER
**Software and algorithms**		

ArcGIS 10.8	ArcGIS software	https://www.arcgis.com/index.html
Minitab 21.1.0	Minitab software	https://www.minitab.com/
Microsoft Excel	Excel	https://www.microsoft.com/microsoft-365/excel

### Experimental model and subject details

#### Study site and environmental conditions

The study was conducted in the Al Sawadi Mangrove Reserve, located 120 km northwest of Muscat in Al Batinah South Governorate (23°45′48.32″N, 57°47′42.56″E) (Figure 2). The 34.3-hectare site, established in 2001 through afforestation efforts, is dominated by *Avicennia marina*, a species adapted to saline conditions (25–48 ppt) and soil pH levels of 7.5–8.5.^14^ This mature mangrove ecosystem serves as a key conservation reference in hyper-arid environments.

Aboveground biomass (AGB) and carbon stock were quantified using an integrated field–remote sensing approach. Destructive sampling provided actual biomass data to calibrate site-specific allometric equations, validated with non-destructive field measurements. Statistical and regression analyses were performed in Minitab 21.1.0, and carbon stock was estimated following IPCC guidelines and converted into CO_2_ equivalents. High-resolution Pléiades Neo imagery (30 cm) was processed to derive vegetation indices, and a Support Vector Machine (SVM) classifier isolated mangrove canopy reflectance. Ground measurements were integrated with spectral indices in ArcGIS Pro using machine learning workflows to produce spatially explicit carbon stock maps. This combined destructive sampling, allometric modeling, and GIS–remote sensing integration provided a robust estimation of mangrove carbon sequestration potential in a hyper-arid environment.

### Method details

#### Study designs

A stratified random sampling design, consistent with Clean Development Mechanism (CDM) methodologies under the UNFCCC,^48^ was employed. A total of 32 sampling plots were established, encompassing 154 randomly selected mangrove trees. The spatial location of each plot was recorded using a high-accuracy RTK GPS device (LT700H tablet with CHCNAV AT312 antenna, ±0.1 m accuracy, China). To ensure consistency, trees exhibiting multi-stemmed structures were measured below the lowest branch bifurcation, following established protocols.^20^^,^^49^ Furthermore, each tree and the corresponding areas occupied by non-woody plants were promptly identified and marked post-measurement to prevent duplication or omission of any tree within the plots.

A representative number of individual mangrove trees from seven distinct species and varying age classes were selected for destructive sampling to ensure comprehensive representation. Prior to harvesting, *in situ* measurements were recorded, including tree height (H), crown diameter (CD), and species-specific wood density (ρ). Stem diameter was measured as the basal diameter at 10 cm above ground level and as the diameter at breast height (DBH) at 1.3 meters for larger trees. Diameters were measured using a 10-meter measuring tape, and tree height was determined using an electronic clinometer.

For buttress biomass estimation, full weight calculation and subsamples were used. All samples were carefully stored in plastic bags to maintain their integrity and prevent any moisture loss. Subsamples from each component were labeled, weighed, and transported to the laboratory at Sultan Qaboos University (SQU). Two samples from the buttress were extracted, including the center and edge portions, dried in an oven, and weighed to estimate the dry weight of the entire buttress. Branches with diameters of 10 to 20 cm were weighed using a scale, and subsamples of mixed branch sizes were dried to determine their dry weight.

Upon arrival at the laboratory, each sample was immediately weighed to record its fresh weight (in grams) and then allowed to air-dry. The samples were subsequently placed in paper bags to facilitate moisture evaporation and transferred to an oven drying room maintained at 80°C for 72–96 hours, following the protocol of in Khalid paper.^50^ During this period, samples were weighed daily until a constant dry weight was achieved, indicating the completion of the drying process. The final dry weight was then used to calculate the dry-to-fresh weight ratio (conversion factor), which was applied to estimate the total dry biomass of each component.

For bole biomass, volume was calculated using measurements of diameter at 5-m intervals and standard geometric formulas, respectively (frustum, cube, cylinder):^51^^,^^52^

$$VOL=\sum \frac{1}{3}\pi L{\left(\frac{D_{t}}{2}\right)}^{2}D_{t}D_{b}{\left(\frac{D_{b}}{2}\right)}^{2}10^{-6}$$

Where VOL is volume (m^3^), L is length (cm), *D*_*t*_ is top diameter of section (cm), *D*_*b*_ is bottom diameter of section (cm).

VOL=L∗W∗H

Where W is Cube width (m), H is Height (m).

$$VOL=\pi *\;r^{2\;}*H$$

Where r is radius (m)

WSG was determined from core samples and compared with literature values, of Standard Operating Procedure (SOP) for Wood Density^52^^,^^53^ and Destructive sampling.^52^^,^^53^^,^^54^^,^^55^^,^^56^

Wood specific gravity (WSG) is typically measured in grams per cubic centimeter (g/cm^3^). A marina’s WSG varies regionally, as indicated by the global wood density database, with reported values ranging from 0.52 in South America, 0.65 in South-east Asia, to 0.732 and 0.689 in Australia.^55^^,^^56^ In this study, WSG was assessed within these ranges to find values closest to actual biomass and for allometric equation calculations. Initially, a WSG value was chosen between the maximum, minimum, and mean value of 0.64775 g/cm^3^ for the allometric equation. However, the actual wood density was determined for this research from the destructive sampling.

The estimation of aboveground biomass and carbon storage ((kg C)/m^2^) within the mangroves of Al Sawadi Reserve involved aggregating the best-fitting models of aboveground carbon. The total carbon stock of the mangrove forest was scaled up by multiplying the overall mean ecosystem carbon stock by the total area occupied by mangroves (34.3 hectares = 0.343 km^2^). To convert this carbon stock into carbon dioxide equivalents (CO_2_ eq), a conversion factor of 44/12 was applied, following established references.^31^^,^^57^^,^^58^

Destructive sampling for aboveground biomass (AGB) estimation is known to be labor-intensive, costly, and environmentally sensitive, particularly in protected ecosystems such as mangroves. Due to these constraints, our destructive sampling was limited to seven representative mangrove trees. This sample size is supported by previous studies showing that even small, well-stratified samples (typically 5–10 trees) can produce reliable allometric models when selected across a range of tree size classes.^58^^,^^59^^,^^60^ In our study, the seven trees were strategically chosen to cover variability in DBH, height, and canopy spread to ensure structural representativeness within ecological and ethical boundaries. This approach aligns with established practices in mangrove and coastal forest biomass research.^61^^,^^62^^,^^63^

Dry biomass estimates were converted to carbon stock using a carbon fraction of 0.47, in line with IPCC guidelines.^33^^,^^34^ Annual carbon sequestration rates were calculated by dividing total carbon mass per hectare by the mean age of the plantation. The resulting values were further converted to CO_2_ equivalents to assess the ecosystem’s carbon mitigation potential.

The crown diameter (CD) of a mangrove shrub was determined, which calculates the average of its widest length (W1) and width (W2), assuming the crown shape resembles a circle or ellipse.

$$CD=\frac{W1+W2}{2\;}$$

The following equation was used to estimate the carbon dioxide equivalents, CO2eq (tons), from the carbon stock^64^:

$$CO2_{eq}=\left(\frac{1}{2}\right)BM\left(\frac{44}{12}\right)\left(\frac{1}{1000}\right)$$

Where BM denotes the total dry biomass of the tree, measured in kilograms (kg). The value 44/12 corresponds to the molecular weight ratio of carbon dioxide (CO_2_) to carbon (C).^57^ The factor 1/1000 is used to convert the result from kilograms to metric tons of CO_2_ equivalents.

#### Remote sensing

To ensure alignment between field and satellite data, we measured 160 individual mangrove trees (non-destructively) for structural parameters (DBH, height, canopy diameter), allowing us to upscale and validate the developed AGB model across a larger dataset. The spatial mismatch between field plot size and satellite resolution was addressed through pixel aggregation and geospatial averaging techniques, ensuring each tree or plot’s footprint corresponded to the satellite pixel scale. This method aligns with established practices for biomass mapping using medium- to high-resolution imagery.^65^^,^^66^

Among the vegetation indices evaluated, our custom-developed metrics—including the Green-Red Vegetation Index (GRVI), Blue Ratio (BR), Red Edge Simple Ratio (RESR), Chlorophyll Red Edge Index (CHLRE), Normalized Difference Vegetation Index (NDVI), and Normalized Difference Red Edge Index (NDRE) exhibited the strongest relationships with field-derived Above-Ground Biomass (AGB). This indicates that the use of our tailored indices contributed meaningfully to the improved model performance, supporting their inclusion over simpler conventional ratios. Model-4, which integrated these indices, produced the highest statistical performance, with an R^2^ value of 0.55 and reduced RMSE compared to models utilizing conventional indices. While the R^2^ indicates a moderate correlation, it is deemed acceptable given the spectral saturation challenges commonly encountered in dense, high-biomass ecosystems such as mangroves. These outcomes are consistent with findings from earlier studies that emphasize the effectiveness of red-edge and near-infrared-sensitive indices for enhancing biomass estimation accuracy in tropical forest environments.^67^

It is important to note that no single metric fully captures model performance. Commonly used validation criteria for biomass estimation include the coefficient of determination (R^2^), total biomass, mean biomass, RMSE, relative RMSE, bias, and relative bias.^68^^,^^69^^,^^70^^,^^71^^,^^72^ High-quality results in one metric (e.g., low RMSE) may not correspond to high scores in others. Furthermore, no universally accepted satellite variables exist across forest types. In such contexts, moderate R^2^ values—such as the 0.55 found in our study—are not uncommon and reflect realistic conditions under spectral saturation and heterogeneity in canopy structure. These findings reinforce the importance of using a combination of tailored indices and site-specific model calibration.

#### Machine learning and GIS integration for biomass estimation

To enhance spatial estimation of carbon stocks, a GIS-integrated remote sensing framework incorporating machine learning was applied. A Support Vector Machine (SVM) classifier effectively distinguished mangrove canopy reflectance from bare soil and water, improving spectral data quality. Zonal statistics were then used to extract vegetation index values for each plot, which were correlated with field-based Aboveground Biomass (AGB) measurements to build predictive carbon models. Automated extraction, regression, and spatial interpolation were performed using Python and R to ensure reproducibility. The RS-Carbon-Seq workflow integrated these outputs to generate high-resolution, pixel-level carbon stock maps, demonstrating the capability of machine learning to capture complex, non-linear relationships between spectral indices and biomass beyond traditional parametric models.^72^^,^^73^ The integration of SVM within this framework has proven particularly effective in classifying high-resolution satellite imagery in vegetation studies.^74^

#### Data sources and uncertainty

The dataset combined field-based measurements from the Al Sawadi Mangrove Reserve with high-resolution Pléiades Neo satellite imagery (30 cm, August 2023) to capture canopy structure and spectral characteristics of *Avicennia marina*. Spatial processing was performed in ArcGIS Pro, and statistical evaluation in Minitab 21.1.0, supported by machine learning routines. While the satellite imagery provided detailed coverage, uncertainties remain due to the limited number of destructively sampled trees available for calibration, potential spectral saturation in dense canopies, and the omission of belowground biomass and soil organic carbon, which may lead to underestimation of total carbon storage. Model evaluation further highlighted variability in predictive accuracy, indicating that results are sensitive to data quality and methodological assumptions. These uncertainties were addressed by validating remote sensing estimates against destructive samples and by contextualizing findings with comparable mangrove studies in arid and hyper-arid environments.

### Quantification and statistical analysis

#### Allometric model development

Five existing allometric models were tested to estimate AGB using independent variables: basal diameter (D), crown diameter (CD), and tree height (H). The best-fitting model was selected based on R^2^, root-mean-square error (RMSE), and significance level (P-value). Subsequently, a log–log regression model was developed for each variable and their combinations.

**Table:** 

List of AGB’s equations tested to fit with Avicennia marina (Mangrove)		
No #	Equation	References
AGB1	0.182∗*D*^2.487^	^75^
AGB2	*e*^(2.474 ∗(*ln*(*D*)-2.757)^ ∗ 1.0787	^41^
AGB3	0.1776∗*D*^2.299^ 200.4∗*D*^2.1^ ∗ 0.001	^22^
AGB4 AGB5	*e*^(-2.281+1.525(*ln*(*D*)+0.831*ln*(*CD*)+0.523*ln*(*H*))^ *e*^(-2.2057+1.741(*ln*(*D*)+0.945*ln*(*CD*))^	^42^
AGB6	1.8247∗*CD*^2^∗*H*	^43^

The performance of these log–log models was evaluated using key statistical indicators, including the coefficient of determination (R^2^), Root Mean Square Error (RMSE), goodness-of-fit, and P-value. Once the most suitable models were identified, they were refined using a comprehensive dataset compiled from approximately 154 individual mangrove trees sampled across 32 plots. For each model, the specific number of trees used to obtain parameters was recorded. One-way Analysis of Variance (ANOVA) was used to compare observed versus predicted AGB values across the models based on R^2^, RMSE, goodness-of-fit, and P-value, offering detailed insights into model behavior. All regression analyses and statistical evaluations were conducted using Minitab software (Minitab LLC, USA).

#### Metric indicators

Model performance was evaluated using multiple statistical indicators: the coefficient of determination (R^2^) to measure correlation strength, MAE (Mean Absolute Error) and RMSE (Root Mean Square Error) to quantify prediction accuracy, MAPE (Mean Absolute Percentage Error) to assess relative error in percentage terms, and Relative Bias to detect systematic over- or underestimation. These complementary metrics ensured a robust assessment of the accuracy and reliability of biomass and carbon stock estimation models.

$$MAE=\frac{\sum_{i=1}^{n}\left(x_{i}-y_{i}\right)}{n}$$

$$RMSE=\sqrt{\frac{\sum_{i=1}^{n}{\left(x_{i}-y_{i}\right)}^{2}}{n}}$$

$$MAPE=\frac{1}{n}\times \sum_{i=1}^{n}\;\left|\frac{x_{i}-y_{i}}{x_{i}}\right|\times 100\%$$

$$RE=\frac{\sum_{i=1}^{n}\left(\;y_{i}-x_{i}\right)}{\sum_{i=1}^{n}x_{i}}\times 100\%$$

Here, xᵢ represents the actual observed value, yᵢ is the predicted value, and n denotes the total number of data points used in the analysis.
